# Understanding the Migration Intention of Psychological Home in Cyberspace

**DOI:** 10.3390/bs13010071

**Published:** 2023-01-13

**Authors:** Sheng-Cheng Lin, Xuan-Ru Zheng

**Affiliations:** Department of Information Management, Tunghai University, Taichung 407224, Taiwan

**Keywords:** psychological home, self-disclosure, psychological ownership, migration, discontinuous usage intention, expectation-disconfirmation theory

## Abstract

The present study views the personal main page on social media as a psychological home in cyberspace, since they have identical characteristics. Many young people share their lives on social media. However, a backlash is triggered among young people when parents start to use social media and attempt to participate in their children’s online activities, causing young users to migrate social media platforms. This study introduced two concepts of psychological home, self-disclosure and psychological ownership, and the research purpose aims to investigate the relationships between self-disclosure, psychological ownership, and migration intention based on the expectation-disconfirmation theory. A survey research method was used in the study. A total of 561 samples were collected through online questionnaires, and SmartPLS 4.0 was applied for analysis. The results reveal that (1) parental involvement in social media has a positive relationship with dissatisfaction; (2) disconfirmation of psychological ownership and disconfirmation of self-disclosure have a negative relationship with dissatisfaction; (3) the greater the users’ dissatisfaction with social media is, the greater the intention to migrate social medias.

## 1. Introduction

### Research Background and Motivation

Social networks emerged with the rapid development of the Internet. For example, Facebook announced in 2022 that Facebook has 2.91 billion monthly active users, and 36.8% of the world’s population use Facebook monthly, making it the most-used social media platform. Humans are increasingly accustomed to using social networks as a channel to share their lives, for example, by sharing travel photos on Facebook, by sharing their creations on Twitter, and by journaling feelings regarding a hectic day on Instagram. As Wang et al. [1] indicated, authentic self-presentation on social networking sites (SNSs) is a common behavior for adolescents.

This study views the personal main page on social media as a psychological home in cyberspace, since they both share many similar characteristics. Psychological home is defined as a sense of belonging, in which self-identity is tied to a particular place [2]. People generally own a main page or homepage on a social media. This virtual space is dedicated to them, and they are granted the rights to arrange exterior and interior settings of this private site. They can choose some cool pictures of worship idols as their wallpapers; list some written words that touched them deeply; welcome visitors with favorite songs; and determine who can visit this virtual place at any time, as some must knock on the door first, and the owner can even just say no to specific slobs. As Sigmon, Whitcomb, and Snyder [2] indicated, a sense of psychological home may encourage a person to modify exterior and interior design that better reflects the identity of them. Moreover, with their specific psychological home in cyberspace, they can develop interpersonal relationships with many people. They can express some viewpoints towards certain topics, they can share something interesting with visitors, and they can post photos of eating delicious foods. Smith [3] addressed how a person’s psychological home could increase by the social support and positive interactions they experience with other people.

In this dedicated virtual place, they can feel safety, warmth, connection, belonging, wellbeing, self-identity, and various positive feeling toward it, just as the sense of psychological home proposed [2]. Since they usually spend much time to make this psychological home in cyberspace prosperous, it is common that people remain on the site and own such a space for a long time. Studies even indicated that some problematic internet and social media use of young people affected their academic success (e.g., [4,5]). However, according to Trivedi [6], young adults between the ages of 20 and 30 were assumed to decrease by 4 per cent since 2019, and the social media users who once used to rely on Facebook switched to other social media platforms. In general, Facebook has been struggling a lot to attract users under the age of 30 since 2012.

This trend has led older people to use social media (e.g., Facebook, Instagram, and LINE) for monitoring their children. Madden [7] noted that older generations are learning to use social media, and the number of people from older generations who use social media doubled year by year. Specifically, the number of people aged 50 to 64 years who use social media such as MySpace, Facebook, or LinkedIn has grown by 88%; the social media usage rate for those more than 65 years of age has grown by as much as 100%. Simonpietri [8] revealed that more people from older generations are using social media as a communication tool. However, older generations are rejected when they start to use social media to communicate with younger generations.

This study postulated that an obvious contradiction exists in the expectations of parents and children for social media use. Turnbull [9] demonstrated that parents and children have different ideas regarding social media (e.g., Facebook). Parents thought that social media use would increase their communication with their children, so that they can express concern for their children and understand their children’s lives and friendships. Nevertheless, children assumed that social media is mainly used to interact with friends. The use of social media to communicate with parents reduces the quality of parent–child communication, so most children avoid online communication with their parents through social media.

Therefore, many social media users complain that parents have invaded their social networks. For younger generations, social networks should be a relaxing space where they can complain, display affection publicly, obtain empathy from people in similar situations, and share opinions. However, young users began to feel monitored after their parents started to use their social networks and to follow or add them as friends on social media. Therefore, parental involvement has become a phenomenon discussed in various forums. Such dissatisfactory experiences by young users are visible in numerous news and social networks. For instance, elder users may criticize or discipline young users by leaving comments publicly under the user’s posts in social networks, complaining about the lavish lifestyle, or redressing photos of a crazy party. Even when young users reject or ignore parents’ friend requests, this may be considered to be unfilial. Numerous younger users stated that they would leave established social media platforms and use alternative ones (e.g., Instagram) that are less likely to be discovered by their parents. Simonpietri [8] observed that young people opt to abandon social media platforms they are currently using and move to alternative ones to avoid attempts at communication by their parents. This serves as a warning to social media developers. The loss of numerous users may cause the decline of a social media platform.

In summary, a phenomenon was observed whereby more parents are using social media for parent–child communication, but this has caused more people from younger generations to migrate social media platforms. This issue is crucial and will remain ever-relevant because human beings are by nature social animals. Younger generations demand that social media constantly updates the innovative information technology used to support interpersonal interaction, whereas the older generations expect an effective communication between parents and children. Social media developers wish to avoid becoming the victims of the parental involvement dilemma. Therefore, this study explored the factors concerning psychological home causing younger generations to migrate social media platforms when the older generations join the one they are currently using.

Because different social media have different functions and characteristics [10], Kaplan and Haenlein [11] used social presence, media richness, self-presentation, and self-disclosure as the theoretical basis for classifying social media into six categories: blogs, content communities, social networking services, virtual social worlds, and virtual game worlds. This study focused on SNSs with a moderate degree of social presence and a high degree of self-disclosure, as represented by Facebook and Instagram. The biggest difference between Facebook and Instagram is that Instagram emphasizes images and telling stories through photos; Facebook centers on text-based content supported by photos.

A person’s psychological home is composed of the senses of secure, warm, and positive feelings where self-expression is permitted and a feeling of belongingness with others both exist [2]. This study therefore extracts self-disclosure and psychological ownership as important constructs from psychological home.

Furthermore, to investigate social media user migration, Kumar et al. [12] defined two types of migration and analyzed the migration of users between seven popular social media sites. Chang et al. [13] developed a scale of virtual migration for social networking sites based on the push, pull, and mooring model. In the same vein, researchers also proposed their scales of social network sites migration according to concepts of the push, pull, and mooring model [14,15,16,17]. Hou [18] explored the switching factors and developed a scale for examining the relationships between those and user’s intentions to switch to different instant messaging media based on the two-factor theoretical perspective.

To investigate the migration intentions from the viewpoint of psychological home, this study proposed a research model based on Oliver’s [19] expectation-disconfirmation theory (EDT) for the comparison of social media platforms and relevant discussion, because EDT is a famous model that addresses the intentions to use a system continually or not. When people decide not to continually participate in a specific social media, they generally do not stop using all kinds of social media, but soon migrate to another social media. Although our view of personal space on social media and psychological home are very similar in several aspects in this study, for convenience and reducing the chance of confusion, this study uses social media to illustrate most concepts below.

## 2. Literature Review

### 2.1. Self-Disclosure

#### 2.1.1. The Concept of Self-Disclosure

Derlega and Grzelak [20] defined self-disclosure as individuals’ reporting and sharing of information relating to themselves, including feelings, thoughts, and experiences. Jourard [21] noted that self-disclosure is the process of presenting oneself to attract attention. Derlega and Margulis [22] further claimed that the five functions of self-disclosure are expression, self-clarification, relationship development, social validation, and social control.

In a modern society with frequent communication, people disclose their personal information and share their thoughts and opinions for communication and trust establishment. This is an essential way of developing interpersonal relationships. In fact, self-disclosure is also regarded as the first step in interpersonal development. In interpersonal interaction, self-disclosure is a communication behavior involving the disclosure of personal information to another party, and the effects depend on the degree of self-disclosure [23].

Ge, Peng, and Chen [24] indicated that self-disclosure emphasizes the reciprocal relationships, and the degree of self-disclosure changes with the degree of disclosure of the other party. As such, self-disclosure is a two-way interaction. Cozby [25] defined self-disclosure as a behavior whereby an individual discloses information relating to himself or herself to another person on the premise of trust, and reciprocal self-disclosure can facilitate a sense of trust, that is, the belief that neither party’s privacy will be violated and that private information will not be used harmfully [26,27].

Scholars have conducted various studies on people in various relationships. Bak et al. [28] claimed that the self-disclosure frequency of social media users tends to be higher in closer relationships. However, Miller et al. [29] argued that, no matter how close a relationship is, people do not reveal all of their private information.

#### 2.1.2. Self-Disclosure on Psychological Home in Cyberspace

Compared to conventional face-to-face communication, online environments have been proven to feature a greater extent of self-disclosure. Moreover, users tend to disclose more information when anonymous [30,31]. This is because they feel safe when they do not see the people they are addressing and can focus on the phrasing of their words and the expression of their emotions. In the study of how Internet users establish interpersonal relationships, Parks and Floyd [32] discovered that people are more likely to speak honestly online than in the real world. This also confirmed the hypothesis of Rheingold [33]—people not only establish substantial relationships through computers, but also express their true feelings more easily because the anonymity of the Internet environment provides a sense of freedom and reduced restraint. Online self-disclosure has been widely discussed and applied by scholars in relation to e-commerce and Internet services [34,35]. Ledbetter [36] also proved that online self-disclosure and social connection are the primary motivators for the use of social media. Therefore, the main initial objective of social media was to establish interpersonal relationships on the Internet. Hagel and Armstrong [37] identified interpersonal relationships as a basic motivation of virtual communities. Among the motivations of Facebook users discussed by Alhabash et al. [38], interpersonal relationships were a key motivation. Self-disclosure is a factor in the establishment of interpersonal relationships because it increases intimacy [39,40].

According to the aforementioned studies, interpersonal relationships are a core motivation for social media use, and self-disclosure is an essential step for people to develop interpersonal relationships in virtual communities. For instance, the wedge model of self-disclosure proposed by Altman and Taylor [41] indicated a causal relationship between self-disclosure and intimacy. Lin and Chu [42] found users of Facebook will have more degrees of self-disclosure when emotionally supported by friends, thereby intimacy emerged. However, it happened when young people posted photos about enjoying an expensive meal, and then was censured by their parents below these photos. Some adolescents expressed agreement with the lifestyle of goblin mode, but were soon criticized by their parents for laziness and passivity just below this comment. These interactions made young people feel embarrassed and could suppress the desire to self-disclose. Therefore, this study inferred that, in the case of parental involvement, users are unable to develop interpersonal relationships effectively because of their inability to disclose themselves, resulting in negative relationships (i.e., behaviors such as discontinuous use or social media platform switching).

### 2.2. Psychological Ownership

#### 2.2.1. The Concept of Psychological Ownership

Since the late 1970s, more companies in western countries have adopted employee stock ownership. Numerous scholars have affirmed that employee stock ownership effectively improves employees’ job performance because formal ownership improves their attitudes and behaviors. However, some have argued that employee ownership is less closely related to job satisfaction and organizational performance than is often claimed. In the study of employee ownership, Pierce et al. [43] proposed the concept of psychological ownership. Van Dyne and Pierce [44] also identified that psychological ownership generates positive attitudes among employees; in addition, Mayhew et al. [45] found that psychological ownership improves employee job satisfaction and organizational commitment.

The fundamental concept of psychological ownership is that human beings possess an innate drive to obtain ownership [46]. The psychology of possession proposed by Furby [47] also constitutes a major theoretical basis for psychological ownership. Objects can be tangible or intangible (e.g., houses, cars, shares, or patents), but the sense of possession is ubiquitous. In addition, Etzioni [48] revealed that psychological ownership can occur either on the basis of legal ownership or in the absence of legal ownership. Therefore, psychological ownership has the characteristics of being semi-subjective, semi-objective, semi-conscious, and semi-realistic. This is a mental state in which an individual feels that a target object is owned. In such states, the individual regards the target object as a medium for self-expression.

Pierce, Kostova, and Dirks [49] claimed that the foundation and motivation for satisfying psychological ownership can be divided into three types, namely, need for territory, efficacy, and self-identity. In the process of satisfying the aforementioned motivations, the psychological ownership of individuals toward a target is gradually established. Pierce, Kostova, and Dirks [50] further proposed that the need for self-identity can be subdivided into three different aspects: coming to know thyself, the expression of self-identity, and the maintenance of continuous self-identity.

Numerous studies on psychological ownership have also been conducted in other fields. For example, Peck and Shu [51] claimed that object touching or object touching prompted by images increases the perception of psychological ownership. Peck et al. [52] found that psychological ownership could enhance stewardship behavior for public goods. Chang, Chiang, and Han [53] also found that psychological ownership provides consumers with a sense of brand ownership, subsequently engendering an altruistic attitude toward the brand.

#### 2.2.2. Psychological Ownership on Psychological Home in Cyberspace

Karahanna et al. [54] unprecedentedly incorporated the concept of psychological ownership into social media, and the findings revealed that social media provides an online platform for users to establish psychological ownership. Moreover, it provides various functions for social media users to create and exchange generated content [11]. Users invest effort and time into creating their own content on social media, which allows them to cultivate a sense of ownership—psychological ownership [54,55]. Zhao et al. [56] suggested that the more effort users dedicate to their personal space, the greater their sense of psychological ownership in social media space becomes, which means that they are willing to devote more time to social media and use it continuously. Furthermore, people generally write blogs to express their opinions to influence others, update their latest activities, seek opinions and feedback from others, write and contemplate, and relax.

Porteous [46] advocated that individuals possess an innate drive to obtain territory, that is, the desire for perceived ownership of a space. Karahanna et al. [54] asserted that people’s need to have their own space is reflected in personal online behavior. Social media provides a personalized page as a virtual personal space, where users can publish basic information, share information, and configure their own personalized interface to satisfy needs for psychological ownership of a place.

White [57] proposed that individuals have an innate motivation for efficacy (i.e., the need to express oneself). Social media meets this demand through four approaches. First, it provides a platform for users to create and exchange opinions with others (e.g., blogging or responding to other users’ messages). Second, efficacy motivation is reinforced through positive feedback from other users, and personal motivation is also enhanced through contribution recognition [58], such as offering likes or leaving approving comments. Third, controlling or influencing an individual generates perception of efficacy, which stems from a sense of career achievement [50]. Social media also provides an excellent environment for influencing or controlling others, such as expressing opinions to influence others’ judgments, managing websites, or leading others in games. Fourth, individuals cultivate perception of efficacy in collaborative projects (e.g., creating or editing Wikipedia entries) by displaying their personal knowledge of a subject [59]. Even if the individual is not the last editor, he or she obtains a sense of participation and contribution.

Individuals gain recognition and social prestige [60] through self-expression and recognition of others [61]. On social media, user-generated content (e.g., photos, videos, or comments) is aimed at sharing the of experiences with others for validation. For example, one can express one’s own views through blogs, sharing experiences of work and life in general, and participating in dialogue and discussion with others [62] to express self-identity. Karahanna et al. [54] proposed that people satisfy their need for self-recognition through social interaction, thereby satisfying their need for self-identity and ultimately exerting a positive effect on psychological ownership. Zhao et al. [56] also proposed that social influence positively affects psychological ownership in the context of social media.

In addition to defining, learning, and expressing oneself, individuals also require a continuous sense of self-identity [49,63]. In the real world, people often retain selected items, such as photos, awards, degrees, and certificates, to maintain their self-identity and connect with their past. Social media can assist people in recovering and maintaining memories. These technologies are generally used to record and store files. Viewing and building individual or group files connects them with memories in people’s lives [64,65,66], and these files allow social media users to express and connect with themselves. In addition, people can also discover old friends or connect with acquaintances through the sharing of videos or photos.

In summary, social media is a favorable platform for the construction of psychological ownership, as proposed by Karahanna et al. [54], who asserted psychological ownership to have a positive relationship with the use of social media. This study inferred that, in the context of parental involvement in social media, young users developed a negative relationship with social media—that is, behaviors such as discontinuous usage or changing social media communities—due to their inability to develop psychological ownership.

### 2.3. Parental Involvement

Laurent and Kapferer [67] suggested that the concept of involvement has been applied in various fields. However, the measurement of involvement has no complete and unified definition because scholars of different research topics have different opinions. Holt et al. [68] defined parental involvement in the context of parents’ knowledge of, active participation in, and level of interest regarding their children’s participation.

As mentioned, older generations have gradually joined the social media spaces originally belonging to younger people as the Internet and social media have developed. Bartholomew et al. [69] mentioned that most parents browse Facebook at least once a day, and it has become a part of their daily lives. In addition, one of the reasons parents use social media is to oversee their children’s lives. In summary, young users experience the impression of being monitored, causing them to discontinue use of their social media after parents join and befriend them on the site. Son and Laura [70] concluded that the behavior of young users on social media when children perceive authoritative or controlling behavior by their parents shows that they are less willing to self-disclose to their parents, which causes intention to discontinue social media usage (hereinafter “discontinuous usage intention”).

The definition of parental involvement was featured in the aforementioned studies and that of Holt et al. [68], and the definition was adapted to the research context of this study, which can be defined as parents’ participation level in the lives of their children on social media. This study also further discussed whether parental involvement produces discontinuous usage intention among children.

### 2.4. Continuous and Discontinuous Usage Intention

Continuous usage of information systems and discontinuous usage of information systems have received increasing attention. More people realized that the value of an information system depends not only on its initial acceptance and adoption but also on long-term continuous use. Therefore, the research focus has been gradually extended from Davis’s [71] technology acceptance model to Bhattacherjee’s [72] post-acceptance model of information systems continuance to explain factors affecting users’ continuation of system use. Studies have verified that satisfaction and perceived usefulness positively affect users’ continuous usage intention. Bhattacherjee affirmed that users’ decisions regarding whether to continue using information systems resemble consumers’ repurchasing decision-making. Later, other scholars used the post-acceptance model of information systems continuance to explain the theoretical basis of the continuous adoption behaviors for various information systems [73,74,75,76,77].

Oliver [19] proposed the EDT, also referred to as expectancy disconfirmation theory. The EDT was originally developed to explore marketing and consumer behavior and was later widely used in consumer behavior research to explore consumer satisfaction and post-purchase behavior [19,78]. According to the EDT framework proposed by Oliver [19], the consumer repurchase intention process is as follows: (1) Consumers establish initial expectations before purchasing a product or service. (2) They accept and start to use the product or service, providing feedback on its performance after a period of time. (3) They evaluate their feelings regarding product performance and compare these with their initial expectations to determine the confirmation level of expectations. (4) They experience satisfaction on the basis of their confirmation level. If the product performance is better than expected, it produces positive disconfirmation and increases satisfaction; otherwise, negative disconfirmation is produced, and consumers are likely to feel dissatisfied. (5) Satisfied consumers generate repurchase intentions, and relatively unsatisfied users discontinue subsequent use.

In the field of information technology, EDT also illustrates how users’ satisfaction with technology depends on how initial expectations compare with product performance [79]. Bhattacherjee [72] combined the EDT framework with a technology acceptance model to propose an expectation-confirmation model (ECM). This model has been widely accepted as an explanation of user satisfaction and the continuous use of information systems [72,73,80]. Bhattacherjee [72] claimed that the decision to continue the use of an information system resembles the consumer repurchase decision. The ECM is established based on three principles: (1) a preliminary acceptance or purchase decision, (2) initial experience of using a product or service, and (3) a decision to continue or discontinue use afterwards. First, any variables preceding acceptance are included in the dimensions of confirmation and satisfaction, so ECM focuses only on the variables after acceptance. Second, because expectations of the use of information systems frequently change over time, ECM measures the expectations after consumption but not before consumption.

Synthesizing both the EDT and ECM, individuals usually have expectations of products or services before use and conduct actual performance evaluations of products or services after use. Both satisfaction and dissatisfaction can reflect the emotional response to the overall experience associated with a certain product or service; dissatisfaction describes negative feelings, such as frustration and discomfort [81]. Numerous studies have found that satisfaction and continuous usage intention have positive effects [82,83]. As such, a positive relationship is expected between dissatisfaction and discontinuous usage intention, and this hypothesis has also been confirmed by several studies. For instance, Luqman et al. [84] discovered that users exhibit stronger intentions of discontinuous use when their dissatisfaction with SNSs is greater.

## 3. Research Design and Methods

### 3.1. Research Model

This study featured an examination of the phenomenon whereby some young social media users terminate social media use and migrate to alternative platforms. This study considered when users applied an account, set user environment, and interacted with other users on specific social media to be establishing a psychological home in cyberspace. Since they usually enjoy a private space to decorate, choices such as color tones, icons, wallpapers, music, avatars representing themselves, and friends lists determine who are welcome to visit any time, and who should send a private message before being permitted to enter. In this dedicated space, they can choose to express their feelings or stories, and share pictures of recent travel.

To conduct the research, this study introduced two important concepts from psychological home. Since there are two common expectations of a psychological home in cyberspace. The first expectation is fulfilling the need for psychological ownership, allowed by the physical amenities inside and outside a house, along with the free will to identify themselves by constructing and decorating their house [2]. Another is fulfilling the need for self-disclosure. As Sigmon et al. [2] asserted, a supportive social network may enhance a person’s freedom of self-expression by establishing a safe place.

Ironically, intruders of psychological home in cyberspace are generally family members from the physical home. It is often when their parents begin to use the same social media platform to communicate with them. This study adopts the EDT proposed by Oliver [19] as a basis for model construction. According to the EDT framework, young users may establish initial expectations to own a private space to share information with friends, and in the chat, anything goes. After their parents start using the social media, they evaluate their influences regarding psychological ownership and self-disclosure and compare these with their initial expectations to determine the disconfirmation level of expectations. Then, they may generate dissatisfaction according to their disconfirmation level. If they feel dissatisfied, they decide to abandon this psychological home in cyberspace and migrate to another social media.

**Figure 1:**
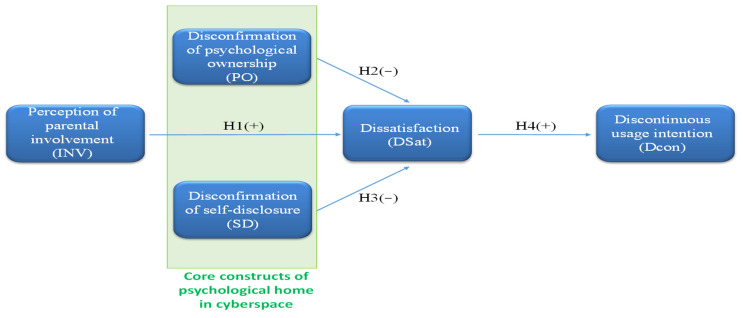
Research Model.

In summary, this study explored the effects of parental involvement in social media on young users’ disconfirmation of self-disclosure and psychological ownership, and whether this involvement further increases users’ dissatisfaction with social media, ultimately leading them to migrate social media platforms. The research model is illustrated in Figure 1.

### 3.2. Research Hypotheses

Turnbull [9] claimed that children avoid communicating with parents on social media because they have different beliefs regarding social media use. Simonpietri [8] found that young users leave the social media platform they are currently using and move to alternative ones to avoid attempts at communication by their parents. West et al. [85] discovered that the reasons for students’ unwillingness to become online friends with their parents are usually related to embarrassment, social norms, and concerns that their parents may be cyber bullied after exposure. The aforementioned literature indicates that users are unable to freely use social media when their parents become involved. According to the EDT, the unfulfilled initial expectations for social media increase dissatisfaction. Accordingly, the following hypothesis was proposed:

**H_1_.**  *Perception of parental involvement has positive influence on dissatisfaction.*

Human beings are born with needs [46]. Etzioni [48] described psychological ownership as a psychological state in which the individual feels that a target object is his or her own. Social media provides each user with an exclusive virtual personal space. Users can set their own themes, music, friends, permissions, and content. Karahanna et al. [54] revealed that social media satisfies users’ needs for psychological ownership. Zhao et al. [56] also noted that people experiencing a higher degree of psychological ownership spend more time using social media.

However, parental involvement may render users unable to develop a sense of psychological ownership for social media. When parents begin to use social media to communicate with them, young users may feel that their ownership of this virtual space has been decreased. High levels of parental involvement in social media may prompt young users to experience a lack of psychological ownership, and they may opt to terminate use of the social media. It just as likely that children have psychological ownership of their own rooms, which they can furnish the room and arrange the contents of their rooms. If their parents frequently enter their rooms, they may feel that their psychological ownership of the room is decreased and intend to move out. According to the EDT, the experience of social media use differs from young users’ initial expectations to when parents invade their spaces, which results in dissatisfaction. Hence, this study assumes that failure to cultivate a sense of psychological ownership and to meet the initial expectations for social media during parental involvement increases dissatisfaction. The following hypothesis was therefore proposed:

**H_2_.**  *Disconfirmation of psychological ownership has positive influence on dissatisfaction.*

Kaplan and Haenlein [11] classified social media as a communication medium with a high level of self-disclosure. Users tend to gain satisfaction through self-disclosure and sharing their experiences with others by using social media (e.g., eating delicious meals, visiting tourist attractions, or expressing emotions). Ledbetter [36] confirmed that online self-disclosure behavior and social connection are the primary factors influencing the use of social media.

Tokić and Pećnik [86] indicated that adolescents perceive their self-disclosures to be influenced by specific parental actions and reactions in disclosure-related situations. For example, they worry that some self-disclosed information (e.g., expensive dining experiences) might be criticized by their parents. Therefore, young users are unable to disclose themselves freely if their parents are also using the same social media. The involvement of parents may render young social media users unable to self-disclose freely on social media. Chege and Obrempong [87] indicated that a higher degree of self-disclosure would contribute to the enhancement of interpersonal relationships within social media. We thus may infer that when people are unable to self-disclose themselves and develop their interpersonal relationships freely on social media, they will feel unsatisfied. High levels of parental involvement in social media may increase the dissatisfaction level of young users, causing them to be unable to disclose themselves and subsequently choose to discontinue the use of social media. Based on the EDT, in situations where parents invade young social media users’ virtual spaces, rendering young social media users unable to disclose themselves without inhibition, users’ initial expectations are unmet, resulting in dissatisfaction. This study inferred that unfulfilled initial expectations due to parental involvement prevent genuine self-disclosure on social media and thus lead to increased dissatisfaction. Accordingly, the following hypothesis was proposed:

**H_3_.**  *Disconfirmation of self-disclosure has positive influence on dissatisfaction.*

According to the EDT, individuals generally have expectations for products or services before using them and conduct actual performance evaluations of products or services after use. Therefore, both satisfaction and dissatisfaction reflect emotional responses to the overall experience of a certain product or service. Specifically, dissatisfaction refers to negative feelings such as frustration and discomfort [81]. Several studies have reported a positive relationship between satisfaction and continuous usage intention [82,83,88]. Oghuma et al. [89] studied the correlation between satisfaction and continuous use of an information system, and the results indicated that users’ continuous usage intention is affected by their degree of satisfaction. Hu et al. [90] also mentioned that satisfaction exerts a substantial influence on users’ continuous usage intention. Lin and Chen [91] also reported that online users’ satisfaction with the website affects their continuous usage intention. As such, dissatisfaction is expected to have a positive relationship with discontinuous usage intention, and this hypothesis has also been confirmed by a few studies. For example, Luqman et al. [84] identified that the discontinuous usage intention is greater when a user’s dissatisfaction with SNSs is greater. Moreover, Kotler [92] noted that users develop a certain degree of satisfaction or dissatisfaction when using website services. If users experience greater satisfaction, their usage intentions are greater and vice versa. Therefore, this study proposed the following hypothesis:

**H_4_.**  *Dissatisfaction has positive influence on discontinuous usage intention.*

### 3.3. Research Design

#### 3.3.1. Questionnaire Design

The questionnaire of this study referred to the definitions in other relevant studies and was modified according to the research context of this study. The questionnaire was divided into six parts. The first part was the basic information of research participants, comprising the most used social media for double checking, sex, age, education, and occupation; the second part was related to parental involvement (three items, modified from Thompson, et al. [93]); the third part inquired about disconfirmation of psychological ownership (nine items, modified from Karahanna et al. [54] and Van Dyne and Pierce [44]); the fourth part surveyed disconfirmation of self-disclosure (nine items, modified from Jourard et al. [21] and Miller et al. [94]); the fifth part measured dissatisfaction level (four items, modified from Bhattacherjee [72]);finally, the sixth part assessed discontinuous usage intention (four items, modified from Maier et al. [95] and Zhang et al. [96]). All variables were measured using the 7-point Likert scale, ranging from strongly disagree (1) to strongly agree (7). To ensure credibility after translation and reduce misunderstanding, the questionnaire was pretested by 20 persons before formal distribution, and wording was revised after participants were questioned regarding their understanding of the items.

#### 3.3.2. Data Collection and Research Participants

The questionnaire was designed in two versions, applicable for Facebook and Instagram users. Items in both questionnaires were the same except that they targeted and distributed to different social media platforms. Questionnaires were created using Google Forms and SurveyCake. There were 21 participants who conducted pre-tests and provided their suggestions for revision to ensure the questions’ meanings and expression were clear before the questionnaires were officially distributed. The pre-test results did not delete any question, but only revised the expressions slightly. Moreover, because this study primarily discussed phenomena caused by social media, the respondents involved were all users of social media such as Facebook and Instagram. Therefore, this study adopted convenience sampling to collect data. The linkages of the online questionnaires were then distributed on social media such as Facebook and Instagram for three weeks. The questionnaire includes a statement that the study involves research and an explanation of the purposes of the research and that it is voluntary, has anonymous participation, has no face-to-face intervention, and that the subjects may discontinue participation at any time. Results of the questionnaire were inputted into an online document in real-time, exported as an Excel file for data preprocessing and data analysis, and stored in an encrypted computer.

#### 3.3.3. Tool for Data Analysis

McDonalds’ ω coefficient provides more realistic estimates of true reliability of scale than Cronbach’s Alpha [97], thus we employed a software named jamovi to calculate McDonalds’ ω coefficient of reliability [98]. Moreover, this study used partial least squares (PLS) as a statistical tool for data analysis, including the validity analysis and the verification of the study models. PLS is an analysis tool of variance-based structural equation modeling (SEM), which is suitable for establishing prediction models and superior to common linear structural relations models (LISREL). PLS has many advantages: there are no requirements for the statistical distribution of data; it does not require too many samples; it can reduce the problem of the collinearity of multiple variables; it can predict exploratory analysis; and it can process questionnaire scales of reflective and formative indexes. Therefore, the present study adopted PLS, conducted analysis with the software SmartPLS 4.0 developed by Ringle, Wende, and Will [99], and calculated the significance of model coefficients by selecting 5000 samples randomly using the bootstrapping algorithm.

## 4. Data Analysis

### 4.1. Descriptive Statistics

A total of 561 questionnaire responses were collected, of which 37 invalid questionnaires (e.g., incomplete questionnaires, repeated submissions, and incorrect answering of reverse questions) were excluded. The number of valid questionnaires was 524; a valid response rate of 93.4%. Facebook and Instagram users accounted for 294 and 230 responses, respectively. The descriptive statistics of participants’ basic data are shown in Table 1.

According to Table 1, the samples contained 248 (47.3%) male respondents and 276 (52.7%) female respondents. The number of female respondents was slightly higher than that of male respondents. In terms of age, the samples were mainly distributed within the 21–30-year age range with 342 people (65.3%), followed by the 31–40-year age range with 79 people (15.1%), and with 67 people (12.8%) younger than 20 years, indicating that most participants were young adults or teenagers. The distributions were akin to the census data of Digital 2021 Taiwan [100]. In terms of academic qualifications, 241 (46.0%) respondents had university or college education, and 187 (35.7%) had graduate-level education or above. Most of the participants, a total of 282 (53.8%), were students.

Trivedi [6] identified that Facebook has been struggling to attract users under the age of 30 since 2012, and young users between the ages of 20 and 30 decreased by 4 percent since 2019. This study focuses on understanding perceptions of young users, and subjects with ages above 30 years old were excluded. Therefore, only 409 young users were investigated, while responses of 115 elder users were used for comparison.

### 4.2. Reliability and Validity Tests of Questionnaire

Prior to structural equation modeling, the reliability and validity of each measurement item required testing.

#### 4.2.1. Reliability Analysis

This study adopted jamovi [98] to calculate McDonalds’ ω coefficient to test the reliability of the questionnaire and examine the internal consistency of the questions designed for each dimension. The reliability analysis results are exhibited in Table 2. The McDonalds’ ω coefficients for all the dimensions of this study were above 0.7, indicating a high level of internal consistency.

#### 4.2.2. Validity Analysis

This study first conducted a structural equation modeling of all users. All three of the parental involvement items were retained because their factor loadings were >0.5. In addition, the composite reliability (CR; 0.893 > 0.7), average variance extracted (AVE; 0.739 > 0.5), and McDonalds’ ω (0.843 > 0.7) for the parental involvement all achieved significance, as shown in Table 3. All nine measurement items for disconfirmation of psychological ownership were retained because their factor loadings were >0.5. The CR (0.955 > 0.7), AVE (0.701 > 0.5), and McDonalds’ ω (0.947 > 0.7) for disconfirmation of psychological ownership all achieved significance. Furthermore, all nine measurement items for disconfirmation of self-disclosure were retained because the factor loadings were >0.5. The CR (0.924 > 0.7), AVE (0.576 > 0.5), and McDonalds’ ω (0.908 > 0.7) for disconfirmation of self-disclosure all achieved significance. All four measurement items for dissatisfaction were retained because their factor loadings were >0.5. The CR (0.959 > 0.7), AVE (0.854 > 0.5), and McDonalds’ ω (0.943 > 0.7) for dissatisfaction all achieved significance. As we can see from Table 2, all four measurement items for discontinuous usage intention were retained because their factor loadings were >0.5. The CR (0.959 > 0.7), AVE (0.855 > 0.5), and McDonalds’ ω (0.944 > 0.7) for discontinuous usage intention all achieved significance. Cross loadings are listed in Table 3.

Pearson correlation coefficient analysis was employed for testing the discriminant validity of each variable. The analysis method involved a comparison of the square root of AVE with the diagonal correlation coefficient. According to Fornell and Larcker [101], the square root of AVE must be greater than the correlation coefficients of other variables to obtain sufficient discriminant validity. In Table 4, the square root of the AVE is between 0.759 and 0.925 for all users, regardless of the platform used, exceeding the correlation coefficients of other variables and compiling with standards.

### 4.3. Research Model Verification

This study adopted SmartPLS 4.0 to verify the model and hypotheses. Specifically, the path coefficient and explanatory power (*R^2^*) of the model were analyzed. The path coefficient represents the level of influence between the variables, and *R^2^* represents the degree to which the independent variables can explain the dependent variable. The verification result of *R^2^* for each variable is shown in Table 5. Both explanatory power of DCON and DSAT are moderate. We can also see the effect size of the model in Table 5. According to Cohen [102], the effect size of DSAT is strong, while INV and SD are moderate, and only PO is weak. Overall, the quality of the model is acceptable.

**Figure 2:**
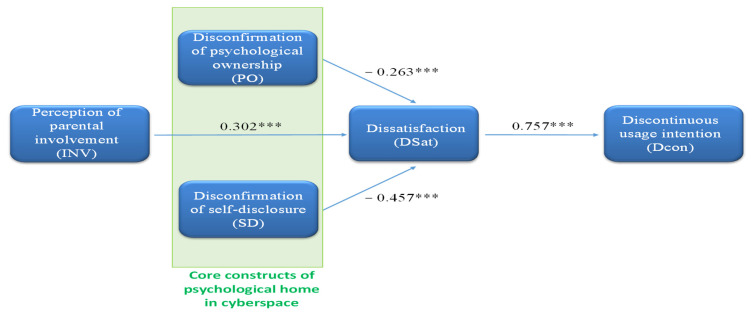
PLS structural model (*** *p*-value < 0.001).

As shown in Table 6 and Figure 2, the path coefficient of H_1_ was 0.302 with a *p*-value = 0.000 < 0.001. The result was significant, and the hypothesis predicted a positive relationship, so H_1_ was supported. The H_2_ path coefficient was −0.263 with a *p*-value of 0.001. There was a negative relationship as predicted. Similarly, H_2_ was supported. Finally, the path coefficients of H_3_ and H_4_ were −0.457 and 0.757 with a *p*-value of 0.000 < 0.001, respectively, so H_3_ and H_4_ were also supported.

### 4.4. Multi-Group Analysis

We conducted two multi-group analyses to answer two questions. Firstly, will different social media contribute to different results? This study examined whether users of Facebook or Instagram exhibit different results. Among 409 young users, there were 223 FB and 186 Instagram users being compared. We can notice the *p*-value of the relationship between perception of parental involvement and dissatisfaction was significant (*p*-value = 0.023 < 0.05). The findings suggest that, except for the significant differences in the parental involvement towards dissatisfaction levels for the users of the two social medias, no other differences between Facebook and Instagram users were identified, indicating that this study has generalizable characteristics. The detailed analysis results are shown in Table 7.

Secondly, another multi-group analysis aimed to understand whether elder users of social media would tell another story when compared with young users. Besides the original 409 young users, we also invited 115 elder users to fill out questionnaires. Table 8 reveals the differences. Again, the relationship between perception of parental involvement and dissatisfaction exhibited discrepancy (*p*-value = 0.018 < 0.05). Other relationships were generally very similar. We will discuss the differences in the next section.

## 5. Discussions and Conclusion

### 5.1. Research Discussions

Using EDT, this study explored the relationship of parental involvement with dissatisfaction and discontinuous usage intention, further introduced from the concepts of psychological home by adding disconfirmation of psychological ownership and disconfirmation of self-disclosure, since we view personal social media and psychological home in cyberspace are nearly identical.

This study revealed the significant and positive relationship of parental involvement with dissatisfaction, indicating that young users’ dissatisfaction increases when their parents use the same social media as they do. According to some brief interviews with subjects, some users reported experiencing the impression that their parents frequently view their social media spaces although they interact with their parents on social media relatively little. As such, the dissatisfaction among young people caused by the impression that parents were viewing their social media was higher than that caused by the number of interactions with parents. Both Facebook and Instagram users experienced increased dissatisfaction when subject to parental involvement, but the extent of dissatisfaction varied. This finding concurs with Simonpietri’s [8] finding that the impression of being monitored causes children to develop the intention to migrate to alternative social media when their parents commence using social media to communicate with them. According to results of this study, a significant and negative relationship was observed between disconfirmation of psychological ownership and dissatisfaction, signifying that the expectations of young social media users after the invasion of their parents into their social media spaces were unmatched or decreased, which also suggested that disconfirmation of psychological ownership is predictive of dissatisfaction. Users possess a sense of ownership of the social media space they are using. In situations where they cannot cultivate a sense of ownership (e.g., due to parental involvement), psychological ownership is naturally affected, which in turn leads to dissatisfaction. For example, individuals feel unable to concentrate on activities when family members or parents enter their rooms uninvited and scrutinize their surroundings, which produces dissatisfaction.

This study also revealed the significant and negative relationship of disconfirmation of self-disclosure with dissatisfaction, meaning that the expectations of young social media users after parental involvement were unmatched or lower, demonstrating that disconfirmation of self-disclosure predicts dissatisfaction. For users, a primary function of social media is self-disclosure with the aim of establishing interpersonal relationships on the Internet. Among the motivators of Facebook users discussed by Alhabash et al. [38], interpersonal relationship is the most essential. Therefore, users may be inferred to experience dissatisfaction with social media when they cannot freely self-disclose and develop interpersonal relationships.

In this study, a significant and positive relationship was observed between dissatisfaction and discontinuous usage intention, implying that dissatisfaction has high predictability for discontinuous usage intention. As in relevant studies, users who are more satisfied with information technology tended to exhibit a higher level of willingness for continuous usage [73,103,104]. Lin and Chen [91] also noted that satisfaction among online users affects their continuous usage intention. In short, social media users are inclined to stop using a particular social media platform or to switch when they experience greater dissatisfaction with the social media they are originally using.

As for multi-group analysis, there was only one discrepancy in perception of parental involvement on dissatisfaction. When they perceived higher parental involvement, Facebook users obviously exhibited dissatisfaction; interestingly, Instagram users did not show the same story. The relationship between perception of parental involvement and dissatisfaction was not significant. We inferred the reason could be that Facebook was developed earlier, and so many young adults are now parents and still use it to communicate with their children. Therefore, young users may perceive higher parental involvement than Instagram. In addition, Prigg [105] indicated that Facebook had much commercial information, such as official pages or formal news, which makes people consider Facebook as a commercial media instead of an interpersonal platform. Moreover, Facebook stresses on the form of expressions by written words with supplementary pictures; on the other hand, Instagram emphasizes using photographs to tell stories. The latter may raise higher interests for young users to join, and thus feel less parental involvement.

The second multi-group analysis examined whether divergence existed between young and elder users. Similarly, the relationship between perception of parental involvement and dissatisfaction were subject to variation. Young users considered higher degree of parental involvement and lower disconfirmation of psychological ownership to make them feel unsatisfied. On the other hand, elder users did not think their involvement would contribute to children’s dissatisfaction at all. They were also unaware of their involvement reducing the sense of psychological ownership of this cyberspace of children, just as many parents arbitrarily enter children’s rooms without preliminary notification. No matter physical or virtual space, maintaining psychological ownership of young users should be important.

### 5.2. Conclusions and Managerial Contribution

In conclusion, most studies on social media use have discussed users’ behavior in social media communities and continuous usage intention. Nevertheless, relatively few studies have explored discontinuous usage or migration intention, which are important for psychological home in cyberspace, since psychological home should be a place that provides safety and privacy from the external world [2]. This study filled the respective gap and tested it with a sample of Facebook and Instagram users. Moreover, this study investigated the negative relationship that social media users’ disconfirmation of psychological ownership and disconfirmation of self-disclosure have with dissatisfaction. Finally, a slight difference between Facebook and Instagram users was found, but, in relation to parental involvement, both types of users demonstrated discontinuous usage intention due to parental involvement.

According to relevant studies and the hypotheses tested in this research, disconfirmation of self-disclosure is a major factor influencing social media use. However, the situation in relation to parental involvement is projected to gradually ameliorate as the parent−child relationship in the east progressively approaches western families comprising a husband–wife axis. For now, social media providers still require approaches to retain young users.

As for managerial contribution, after determining the responses of young social media users to parental involvement and the relevant factors that are involved, social media companies can modify their products accordingly, for instance, by reducing users’ impression that they are being monitored by their parents on social media, and thus may reduce the migration intentions of social medias. This is very important because parents always want to care about their children through certain vehicles, while children always try not to be monitored and seek more freedom. As the Internet and social media continue to develop in the future, the trend for social media use will increase among older generations, and communication with their children through social media will inevitably continue.

### 5.3. Research Limitations and Recommendations for Subsequent Studies

There are some research limitations. This study investigated only users of Facebook and Instagram communities, which may affect the generalization of the results. Moreover, this study adopted a cross-sectional design, which represented the statuses of social media users only at a particular time. More valuable conclusions may be obtained with a longitudinal cross-sectional study.

For future research, this study did not take into consideration parenting styles. Because the parent–child relationship in the east is beginning to more closely resemble western families comprising a husband–wife axis, subsequent studies are recommended to investigate whether parental involvement will continue to cause dissatisfaction among young social media users regardless of the changes toward a more relaxed parenting culture or not. Furthermore, subsequent studies are recommended to collect questionnaires at multiple time points to investigate whether time affects users’ perceptions of parental involvement. Moreover, this study also revealed that users of Facebook and Instagram exhibit differences in their dissatisfaction subject to parental involvement. Future researchers can further discuss what characteristics of various social medias contribute to the differences.

## Figures and Tables

**Table 1:** Descriptive statistics.

Characteristic	Category	Sample Size	Percentage
Gender	Male	248	47.3%
	Female	276	52.7%
	Total	524	100%
Age	Below 20	67	12.8%
	21~30	342	65.2%
	31~40	79	15.1%
	41~50	23	4.4%
	Above 51	13	2.5%
	Total	524	100%
Education	Junior High School or Below	11	2.1%
	Senior High/ Vocational School	85	16.2%
	University/College	241	46.0%
	Graduate School or Above	187	35.7%
	Total	524	100%
Occupation	Student	282	53.8%
	Manufacture Industry	31	5.8%
	Service Industry	98	18.7%
	Finance/Insurance Industry	35	6.7%
	Technology Industry	45	8.6%
	Public Service	27	5.2%
	Others	6	1.2%
	Total	524	100%

**Table 2:** Results of reliability and validity analysis.

Constructs	ITEMs	Factors Loadings (>0.5)	CR (>0.7)	AVE (>0.5)	McDonalds’ ω (>0.7)
Perception of parental involvement (INV)	INV 1	0.694	0.893	0.739	0.843
	INV 2	0.928			
	INV 3	0.935			
Disconfirmation of psychological ownership (PO)	PO 1	0.788	0.955	0.701	0.947
	PO 2	0.823			
	PO 3	0.803			
	PO 4	0.900			
	PO 5	0.876			
	PO 6	0.884			
	PO 7	0.854			
	PO 8	0.817			
	PO 9	0.778			
Disconfirmation of self-disclosure (SD)	SD 1	0.787	0.924	0.576	0.908
	SD 2	0.736			
	SD 3	0.665			
	SD 4	0.842			
	SD 5	0.758			
	SD 6	0.814			
	SD 7	0.807			
	SD 8	0.675			
	SD 9	0.727			
Dissatisfaction (DSat)	DSat 1	0.934	0.959	0.854	0.943
	DSat 2	0.925			
	DSat 3	0.894			
	DSat 4	0.943			
Discontinuous usage intention (DCon)	DCon1	0.928	0.959	0.855	0.944
	DCon 2	0.916			
	DCon 3	0.928			
	DCon 4	0.927			

**Table 3:** Cross loadings.

	DCon	DSat	INV	PO	SD
DCon1	**0.928**	0.699	0.150	−0.607	−0.654
DCon2	**0.916**	0.700	0.245	−0.593	−0.669
DCon3	**0.928**	0.703	0.117	−0.627	−0.678
DCon4	**0.927**	0.699	0.099	−0.613	−0.673
DSat1	0.695	**0.934**	0.193	−0.513	−0.563
DSat2	0.689	**0.925**	0.148	−0.537	−0.613
DSat3	0.718	**0.894**	0.226	−0.513	−0.590
DSat4	0.696	**0.943**	0.228	−0.520	−0.596
INV1	0.155	0.137	**0.694**	0.098	0.054
INV2	0.134	0.187	**0.928**	0.196	0.100
INV3	0.146	0.221	**0.935**	0.183	0.052
PO1	−0.420	−0.338	0.342	**0.788**	0.550
PO2	−0.526	−0.380	0.227	**0.823**	0.591
PO3	−0.597	−0.547	−0.104	**0.803**	0.686
PO4	−0.637	−0.581	0.075	**0.900**	0.705
PO5	−0.558	−0.465	0.222	**0.876**	0.676
PO6	−0.637	−0.554	0.077	**0.884**	0.733
PO7	−0.576	−0.530	0.085	**0.854**	0.697
PO8	−0.468	−0.368	0.405	**0.817**	0.633
PO9	−0.443	−0.332	0.392	**0.778**	0.573
SD1	−0.640	−0.558	0.000	0.743	**0.787**
SD2	−0.468	−0.509	−0.025	0.461	**0.736**
SD3	−0.388	−0.414	0.000	0.386	**0.665**
SD4	−0.579	−0.512	0.105	0.669	**0.842**
SD5	−0.528	−0.383	0.266	0.665	**0.758**
SD6	−0.597	−0.491	0.207	0.697	**0.814**
SD7	−0.560	−0.498	−0.012	0.524	**0.807**
SD8	−0.546	−0.381	0.202	0.584	**0.675**
SD9	−0.597	−0.554	−0.101	0.606	**0.727**

Note: 1. INV = perception of parental involvement; PO = disconfirmation of psychological ownership; SD = disconfirmation of self-disclosure; DSat = dissatisfaction; DCon = discontinuous usage intention. 2. Bold on number denotes a high correlation between items of the same construct.

**Table 4:** Results of correlation analysis.

	DCon	DSat	INV	PO	SD
DCon	**0.925**				
DSat	0.757	**0.924**			
INV	0.165	0.216	**0.860**		
PO	−0.659	−0.564	0.191	**0.837**	
SD	−0.723	−0.639	0.079	0.785	**0.759**

Note: 1. INV = perception of parental involvement; PO = disconfirmation of psychological ownership; SD = disconfirmation of self-disclosure; DSat = dissatisfaction; DCon = discontinuous usage intention. 2. Bold on number denotes the square root of AVE.

**Table 5:** Explanatory power (*R^2^*) and effect size of the model.

Constructs	R-Square		Effect Size (f^2^)			
	R-Square	R-Square Adjusted	DSat	INV	PO	SD
**DCon**	0.573	0.572	1.344	0.175	0.051	0.160
**DSat**	0.505	0.502				

Note: INV = perception of parental involvement; PO = disconfirmation of psychological ownership; SD = disconfirmation of self-disclosure; DSat = dissatisfaction; DCon = discontinuous usage intention.

**Table 6:** Path coefficients of the model.

	Original Sample (O)	Sample Mean (M)	Standard Deviation (STDEV)	*t* Statistics (|O/STDEV|)	*p*-Values
INV → DSat	0.302	0.304	0.037	8.134	0.000 ***
PO → DSat	−0.263	−0.260	0.078	3.366	0.001 ***
SD → DSat	−0.457	−0.460	0.075	6.096	0.000 ***
DSat → DCon	0.757	0.758	0.030	25.645	0.000 ***

Note: INV = perception of parental involvement; PO = disconfirmation of psychological ownership; SD = disconfirmation of self-disclosure; DSat = dissatisfaction; DCon = discontinuous usage intention. *** *p*-value < 0.001.

**Table 7:** Multi-group analysis of Facebook and Instagram users.

	FB				IG				Difference	
	Original	STDEV	*t* Value	*p*-Value	Original	STDEV	*t* Value	*p*-Value	Coefficients Difference (FB-IG)	2-Tailed *p*-Value
INV → DSat	0.338	0.051	6.575	0.000	0.139	0.080	1.751	0.080	0.198	0.023 *
PO → DSat	−0.239	0.116	2.062	0.039	−0.302	0.116	2.590	0.010	0.063	0.700
SD → DSat	−0.391	0.105	3.719	0.000	−0.492	0.111	4.439	0.000	0.101	0.506
DSat → DCon	0.771	0.039	19.779	0.000	0.742	0.045	16.532	0.000	0.029	0.625

Note: INV = perception of parental involvement; PO = disconfirmation of psychological ownership; SD = disconfirmation of self-disclosure; DSat = dissatisfaction; DCon = discontinuous usage intention. * *p*-value < 0.05.

**Table 8:** Multi-group analysis of young and elder users.

	Young				Elder				Difference	
	Original	STDEV	*t* Value	*p*-Value	Original	STDEV	*t* Value	*p*-Value	Coefficients Difference (Young-Elder)	2-Tailed *p*-Value
INV → Dsat	0.302	0.037	8.134	0.000	0.029	0.096	0.300	0.764	0.273	0.018 *
PO → Dsat	−0.263	0.078	3.366	0.001	−0.138	0.161	0.853	0.394	−0.125	0.485
SD → Dsat	−0.457	0.075	6.096	0.000	−0.356	0.146	2.445	0.015	−0.101	0.538
DSat → Dcon	0.757	0.030	25.645	0.000	0.746	0.057	13.131	0.000	0.011	0.895

Note: INV = perception of parental involvement; PO = disconfirmation of psychological ownership; SD = disconfirmation of self-disclosure; DSat = dissatisfaction; DCon = discontinuous usage intention. * *p*-value < 0.05.

## Data Availability

Research data available upon request.
